# Cystic angiomatosis in children: clinical experience and review of literature

**DOI:** 10.1186/s12957-022-02864-z

**Published:** 2022-12-09

**Authors:** Wen Chao Li, Li Liu, Zhen Dong Wang, Hui Chen, Guang Liu, Zhi Chun Feng

**Affiliations:** 1 Department of Pediatrics, The Seventh Medical Center of Chinese People’s Liberation Army General Hospital, No. 5 Nanmen Cang Hutong, Dongcheng District, Beijing, People’s Republic of China; 2 Department of Pathology, The Seventh Medical Center of Chinese People’s Liberation Army General Hospital, No. 5 Nanmen Cang Hutong, Dongcheng District, Beijing, People’s Republic of China

**Keywords:** Cystic angiomatosis, Multiple osteolytic lesions, Child, Oncology

## Abstract

**Background:**

Cystic angiomatosis is a rare benign disease manifesting as multiple lytic and sclerotic bone lesions, described as the proliferation of vascular and lymphatic channels lined by a single layer of endothelial cells. However, the potential pathogenetic mechanism of the disease still remains unknown. Here, we reported a case of cystic angiomatosis with multifocal bone lesion evaluated by whole exome sequencing.

**Case description:**

In this presentation, we reported a case of an 11-year-old boy with pain in his chest. Computed tomography (CT) revealed the multiple lytic of the bone in the ribs, clavicle, vertebra thoracalis, skull, mandibula, shoulder blade, etc. The blood test showed ALP to be 393U/L and VEGF to be 287.26 pg/ml. The patient was performed with an open biopsy in the ribs and was diagnosed with cystic angiomatosis. Besides, the whole exome sequencing reported the single-nucleotide substitutions in the coding region of BRIP1, CHEK2, GRM4, and MUC16. Then, the upregulated genes involved CASC15, CENPF, ABCA13, ALK, BLM, and FGFR3.

**Conclusions:**

In this article, we report a rare case of cystic angiomatosis in a child with abnormal VEGF and ALP reported by peripheral blood examination. The whole exome sequencing could provide the reference for the potential molecular mechanism in the diagnosis and treatment of cystic angiomatosis.

## Introduction

Cystic angiomatosis is a rare, benign disease characterized by disseminated multifocal hemangiomatous and/or lymphangiomatous lesions of the skeleton, which was firstly reported by Jacobs and Kimmelstiel in 1953 [1]. The disease mainly affects axial or appendicular bone involving the pelvis, long bones, and shoulder girdles [2], as well as the visceral organ of the lungs, liver, or spleen [3]. The radiological manifestation of systemic cystic angiomatosis is generally confused with secondary malignant neoplasia, Langerhans cell histiocytosis, metastatic cancer, or skeletal involvement in other malignancies [2, 4, 5], which are asymptomatic in most cases. When the bone is affected by pathological fracture or adjacent soft tissue injury, the clinical symptom such as pain, swelling of the affected area, or function limitation could occur in patients [6]. The neurological symptom could present in the vertebral localization or skull lesion [7]. The diagnosis of cystic angiomatosis is difficult, as the pathologic result is generally unpredictable in some cases. Despite its relation with the disorder of vascular malformations demonstrated by the previous literature [3], the exact pathogenetic mechanism of this disease still remains unclear.

Hence, we reported a case of an 11-year-old boy manifesting chest pain with typical multifocal skeletal involvement, which was diagnosed as cystic angiomatosis by repeatedly open biopsy. Afterwards, the whole exome sequencing was performed, so as to explore the potential molecular mechanism in the development of cystic angiomatosis.

## Case presentation

**Fig. 1:**
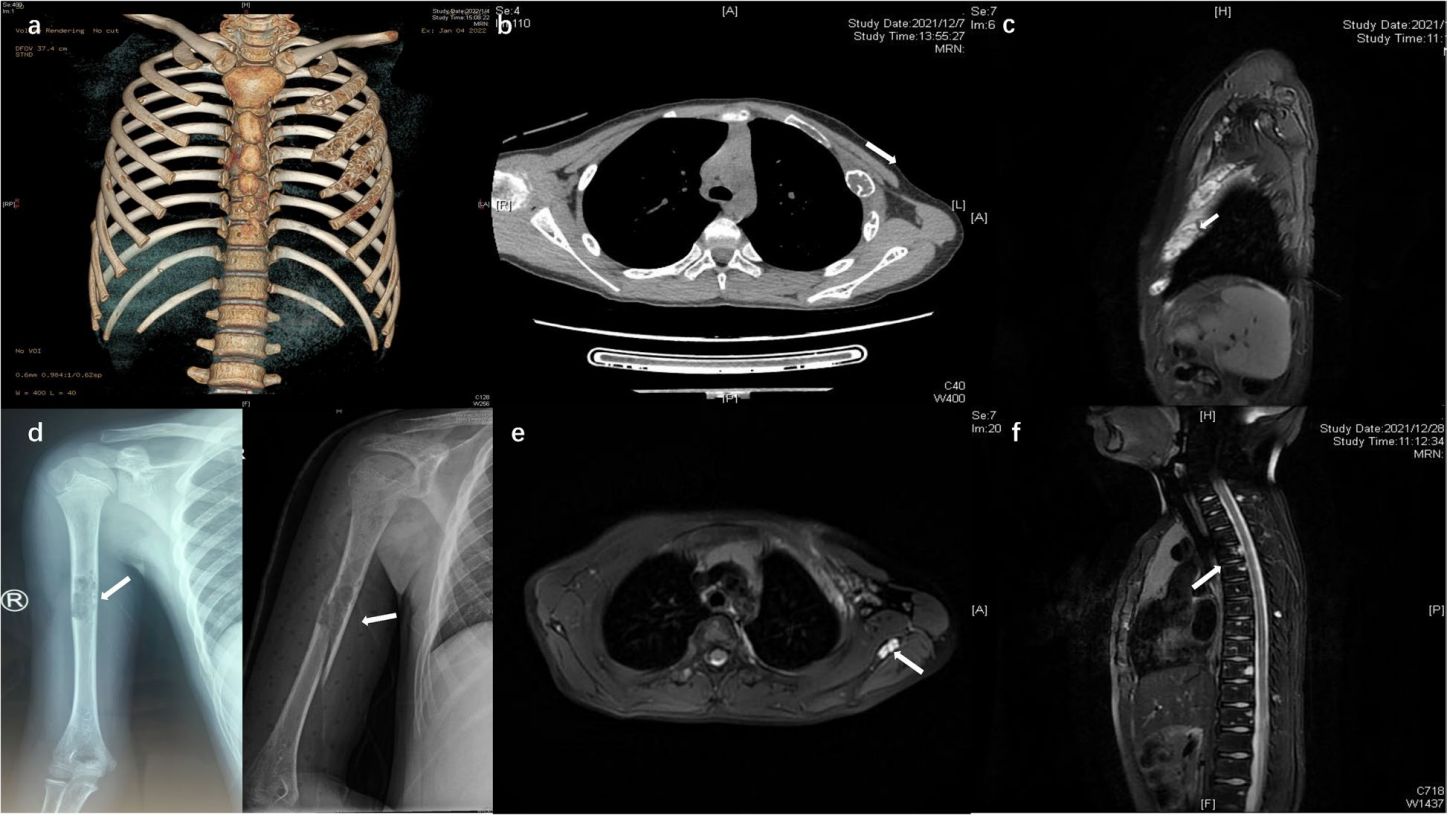
The multiple lesion bone in the left ribs in CT (**a**, **b**) and MRI (**c**). The lesion in the right humerus and pathological fracture (**d**). MRI scan (T2) showed multiple lytic areas in the scapula (**e**) and vertebral bodies (**f**)

An 11-year-old boy was referred to pediatric orthopedics due to pain in the chest. There reported no remarkable family history or trauma history. Radiological examination showed multiple cystic lesions in the left ribs, right humerus, and proximal left radius, accompanied by the fracture of the right lib. The left ribs exhibited multiple lytic lesions with the attenuation of cortical bone (Fig. 1). Computed tomography (CT) revealed multiple lytic of the bone in the ribs, right clavicle, vertebra thoracalis, mandibula, and shoulder blade without tissue swelling. The biopsy surgery was performed for the lesion bone of the left rib and right humerus, which, however, regrettably reported no definite pathological result. Unfortunately, the boy exhibited a pathological fracture in the affected area of the humerus after surgery and was treated by a brace of the upper limb.

**Fig. 2:**
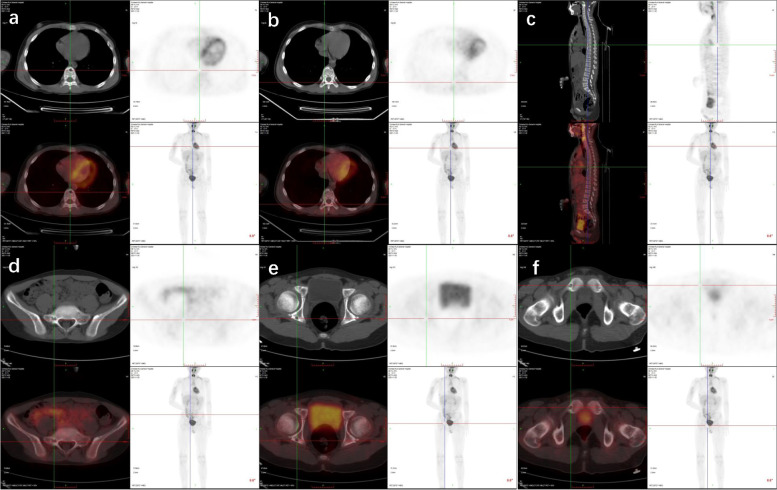
PET showed that avid radioisotope uptake in areas involving vertebra thoracalis (**a**, **b**, and **c**), rumpbone (**d**), acetabular bone (**e**), and pubic bone (**f**)

**Fig. 3:**
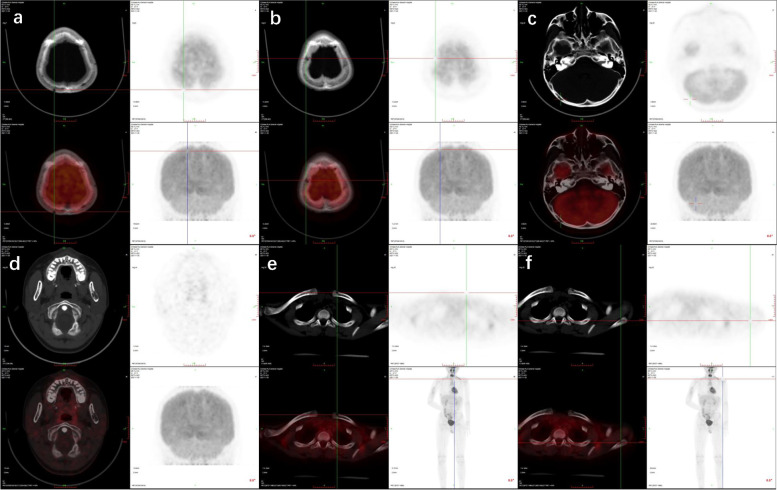
PET showed that avid radioisotope uptake in areas involving the skull (**a**, **b**, and **c**), mandibula (**d**), clavicle (**e**), and shoulder blade (**f**)

**Fig. 4:**
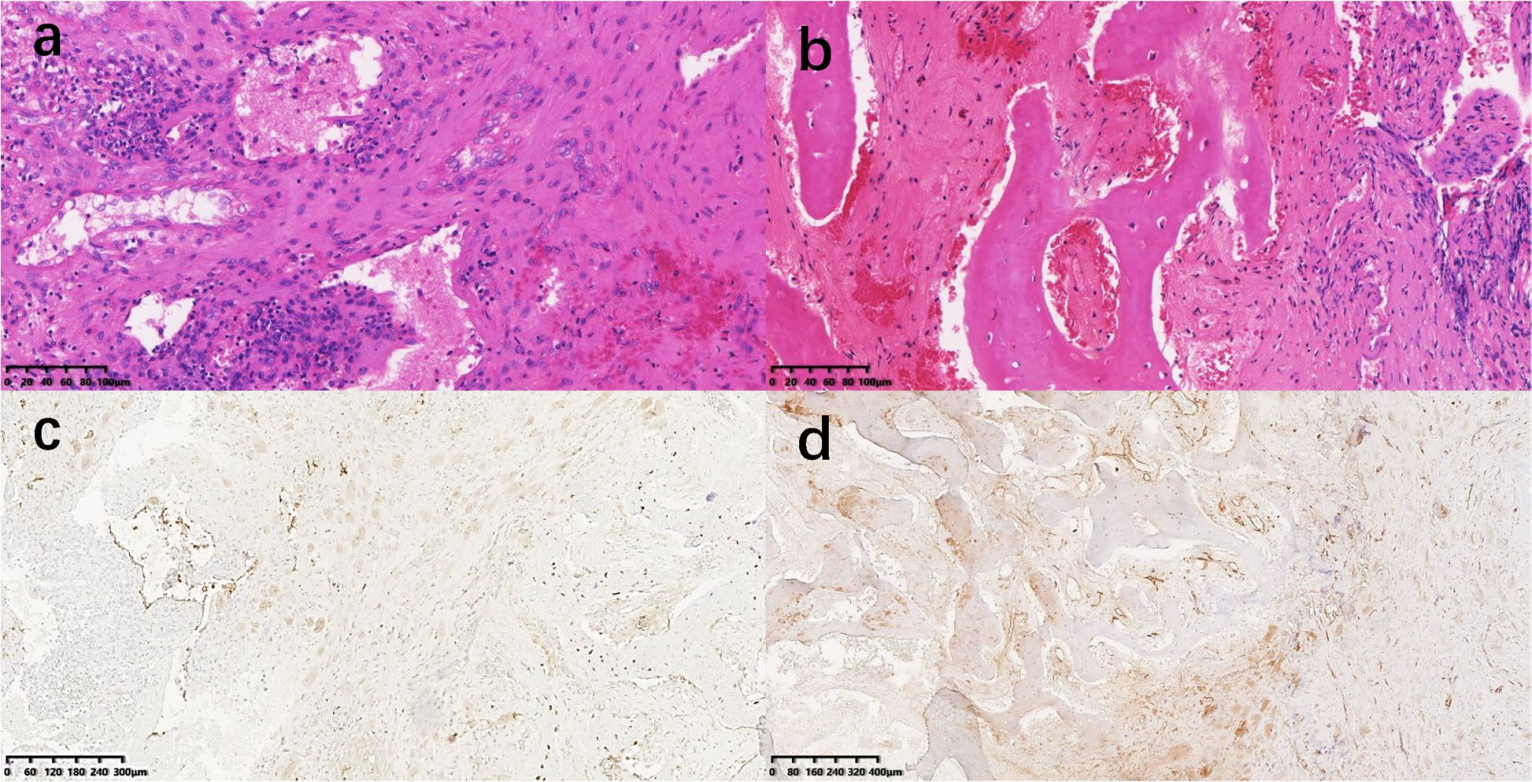
Histopathological examination showed the fibrous connective tissue, dilated blood vessels, and dilated lymphatic vessels around the trabecular bone (**a**). There were positives of CD31 (**b**) and D2-40 (**c**) in immunohistochemical staining

The boy was later referred to our hospital. Routine blood results showed alkaline phosphatase (ALP) to be 393U/L, with a normal of 20–110 U/L and vascular endothelial growth factor (VEGF) 287.26 pg/ml, with a normal of 0–142 pg/ml. The results of positron emission computed tomography (PET) showed avid radioisotope uptake in affected areas covering vertebra thoracalis, rumpbone, acetabular bone, pubic bone, skull, mandibula, clavicle, and shoulder blade (Figs. 2 and 3). The open biopsy was additionally performed on the left ribs. The cortex of the ribs was thinned with the cortex surface of kermesinus, of which the inside space was dilated, filled with the hydatid fluid of erythrina. The pathological examination result of the second rib showed collagen sclerosis in hyperplastic fiber connective tissue, lymphocyte infiltration, and partial foam-like tissue cells, without obvious eosinophile granulocyte in the lesion. Besides, the affected area in the third rib exhibited fibrous connective tissue and dilated blood vessels with bleeding in the trabecular bone, and the dilated lymphatic vessels with lymphocytes (Fig. 4). There involved CD31(+), D2-40(+), SATB2(+), CD20(+), SMA (+), S-100 (−), and BRAF-V600E (−) in immunohistochemical staining. There was no pathological examination evidence of malignancy. The final diagnosis was generalized as cystic angiomatosis.

**Fig. 5:**
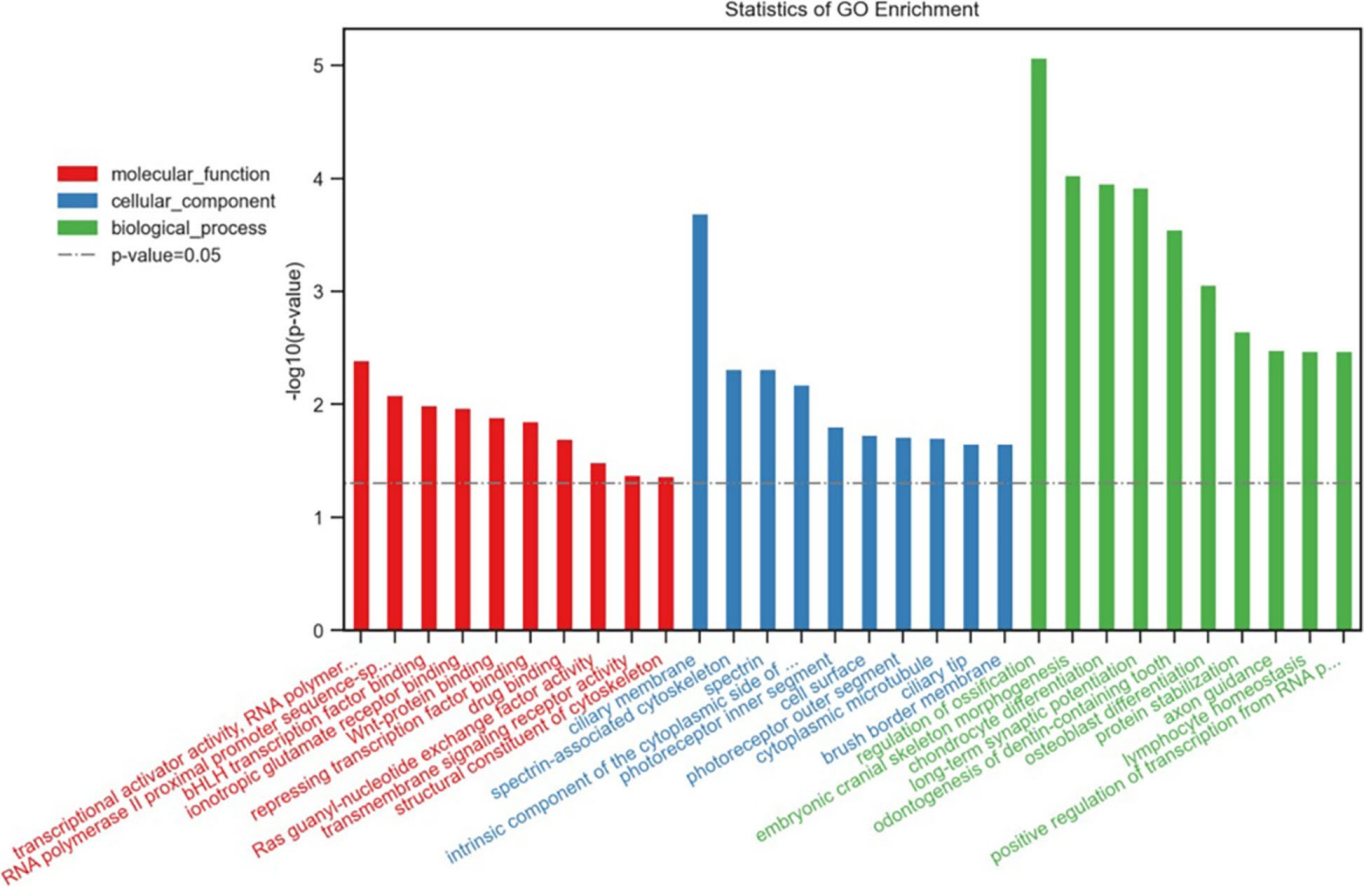
GO functional enrichment analysis revealed that BP, CC, and MF of the upregulated gene

**Fig. 6:**
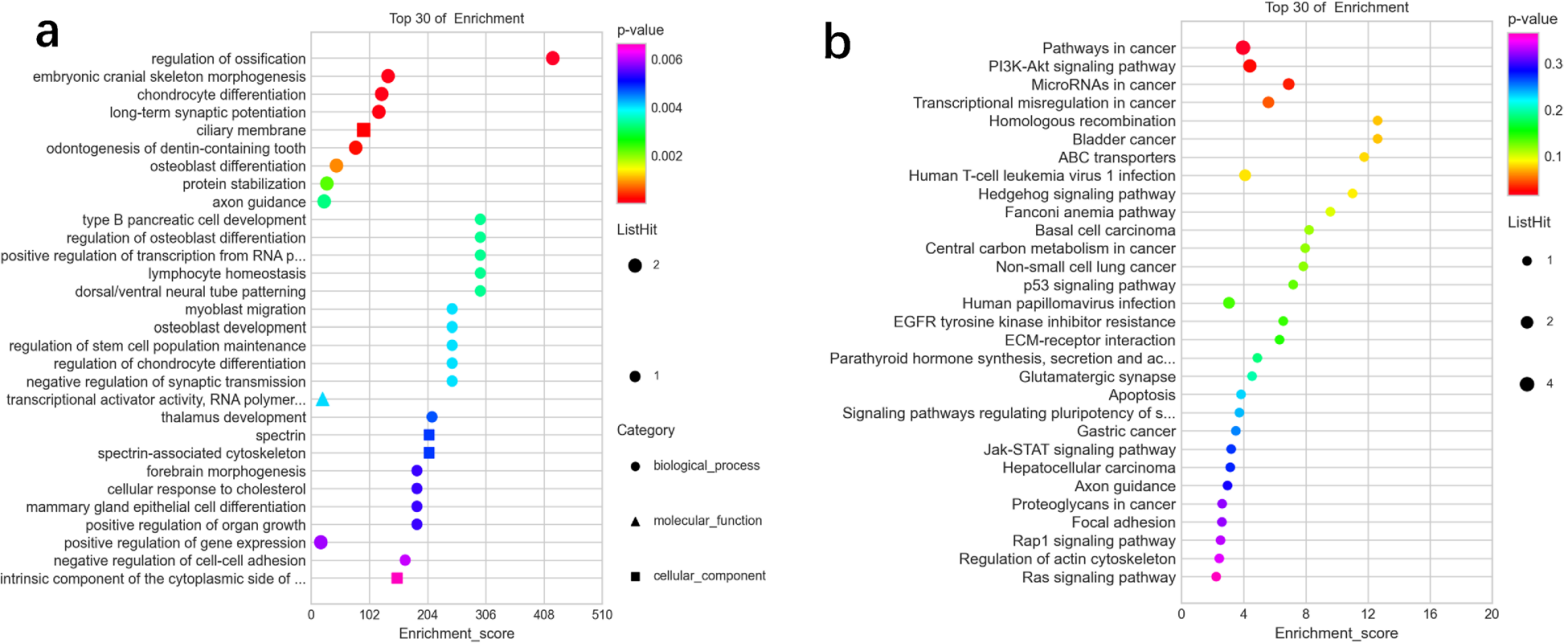
The bubble diagram of TOP30 DEGs in GO function enrichment (**a**) and KEGG function enrichment (**b**)

According to whole exome sequencing, there were some single-nucleotide substitutions in the coding region covering BRIP1 (c.2071A>T (p.I691F) 8.3%), CHEK2 (c.1555C>G (p.R519G) 2.7%; c.1525C> T (p.P509S) 1.8%), GRM4 (c.278G>C (p.R93P) 5.4%), and MUC16 (c.39613C>T (p.P13205S) 3.2%; c.39611A>C (p.K13204T) 1.3%). Besides, the upregulated genes involved CASC15, CENPF, ABCA13, ALK, BLM, and FGFR3. Gene Ontology (GO) functional enrichment analysis revealed the biological process (BP) of an upregulated gene involving regulation of ossification, embryonic cranial skeleton morphogenesis, and chondrocyte differentiation (Fig. 5). Cellular component (CC) analysis revealed that upregulated DEGs were primarily enriched in the ciliary membrane, spectrin-associated cytoskeleton, and spectrin. Molecular function (MF) of upregulated DEGs were demonstrated to cover bHLH transcription factor binding, ionotropic glutamate receptor binding, and Wnt-protein binding (Fig. 6). According to KEGG pathway enrichment analysis, upregulated DEGs were mainly enriched in pathways involving pathways in cancer, PI3K-Akt signaling pathway, MicroRNAs in cancer, and transcriptional misregulation in cancer (*P*<0.05).

## Discussion

Cystic angiomatosis is a rare disease characterized by multifocal bony cysts with a honeycombed appearance and thin-walled blood vessel proliferation with bone destruction, where bone trabecula under the cortex is gradually replaced by hemangiomatous or lymph angiomatous tissue. The first decades of life, particularly puberty, is one of the periods with the highest rates of cystic angiomatosis, and ages higher than 60 years may be the second stage for occurrence peak [3]. The clinical symptoms vary from accidental findings of radiograph to pathologic fracture or skeletal abnormalities of slow progression, or manifesting as rare severe visceral lymphangiomatosis, especially for the lung, liver, or spleen [6]. Radiograph imaging reports multifocal and skeletal intramedullary cysts, with a thinned and relatively well-preserved bony cortex, without a peripheral soft tissue involvement and periosteal reaction [7].

The cystic angiomatosis is typically characterized as multifocal intramedullary skeletal cysts with relatively well-preserved cortical bone, without periosteal reaction and peripheral involvement of soft tissues in the lesion of the bone, while metastasis, multiple myeloma, or other malignant diseases often involve peripheral or tissue reaction [3, 4]. Bone cysts are oriented along the long axis of the bone developing a sclerotic peripheral ring. Cystic angiomatosis and Gorham-Stout disease (GSD) present similar features in the destruction and resorption of the bone [4, 8]. However, cystic angiomatosis exhibits sclerosis appearance in the margin of cysts and sclerosing lesions rather than osteolysis in the affected bone, which is the critical manifestation in GSD. Most previous studies demonstrated that GSD could elicit progressive massive osteolysis, resulting in cortical bone loss, causing severe deformations and disability, whereas cystic angiomatosis-induced medullary cavity without progress of disease [9], which demonstrates that cystic angiomatosis usually has a better prognosis than GSD [10].

Bone biopsy is a standard method in the diagnosis of cystic angiomatosis. However, previous studies have usually argued that histological diagnosis is significantly difficult in some diseases, requiring repeated bone biopsy for final diagnosis [5]. In our study, the first biopsy surgery was performed in other hospitals, which, as a result of an undefined diagnosis, caused the lesion of the lib and humerus regrettably filled with bone fluid. In the second bone biopsy, we found bleeding in the trabecular bone from fibrous connective tissue and dilated blood vessels, lymphocytes, and cystic wall of endothelial lining in the dilated lymphatic vessels, where the cystic wall was reported by most literature [6, 11]. We considered these as consecutive phases in the development process of cystic angiomatosis, which are different histological results. Thus, the different radiological performances in the content of bone cystic should be evaluated before the bone biopsy.

There showed an increase in ALP and bone marker osteoprotegerin, osteopenia, and interleukin-6 in cystic angiomatosis [7], where ALP was 393 U/L with the normal of 20–110 U/L, and VEGF of 287.26 pg/ml with a normal of 0–142 pg/ml, which serves as a growth factor with critical pro-angiogenic activity, promoting the vascular permeability and cell migration. Besides, VEGF and their endothelial tyrosine kinase receptors are involved in vasculogenesis, angiogenesis, and lymphangiogenesis, promoting the angiogenic pathway through signaling with VEGFR-2. VEGFR1 and VEGFR2 are mainly focused on vascular endothelial cells and VEGFR-3 on lymphangiogenesis especially [12]. Histological examination shows that the vessels of vascular or lymphangiomatous are involved in VEGF and podoplanin. CD31 is another notable marker of cystic angiomatosis, indicating a hematopoietic lineage. In our case, CD31 also exhibited positive in the immunohistochemistry, indicating lymphatic or angiomatous proliferations. The current medical treatment for cystic angiomatosis mainly refers to bisphosphonates, interferon-a, calcitonin propranolol, steroids, alpha-interferon, surgery, and radiation therapy [5, 7]. Thus, anti-VEGF or anti-VEGFR therapy may have the potential capability in blocking angiogenesis or pathological processes in cystic angiomatosis.

In the whole exome sequencing, we found some single-nucleotide substitutions in the coding region including BRIP1, CHEK2, GRM4, and MUC16. Besides, the upregulated genes involved CASC15, CENPF, ABCA13, ALK, BLM, and FGFR3, which may indicate the pathogenesis of cystic angiomatosis to some extent. BRIP1 pathogenic germline variants may play a causal role in CRC as moderate cancer susceptibility alleles, with association to hereditary CRC predisposition, which could elevate the risk of developing hereditary ovarian cancer [13]. CASC15 is involved in the manipulation of biological processes in various diseases, which could be a new potential biological therapeutic target [14], as its abnormal down-expression has been found in ovarian cancer, glioma, and neuroblastoma [15]. CENPF plays a key role in the regulation of the cell cycle [16], of which the levels contributed to increased cell proliferation by mediating apoptosis and cell cycle in osteosarcoma with a poor prognosis of osteosarcoma [17]. GO functional enrichment analysis revealed that the BPs of upregulated genes covered regulation of ossification, embryonic cranial skeleton morphogenesis, and chondrocyte differentiation. CC analysis revealed the primary enrichment of upregulated DEGs in the ciliary membrane, spectrin-associated cytoskeleton, and spectrin. The MFs of upregulated DEGs were demonstrated to participate in bHLH transcription factor binding, ionotropic glutamate receptor binding, and Wnt-protein binding.

## Conclusions

Multiple lytic bone lesions without periosteal reaction and involvement of soft tissues in children require to be evaluated in the diagnosis of cystic angiomatosis, which can be promoted by peripheral blood examination including VEGF or ALP. The differential radiological performance related to the content of the cystic should be considered before the bone biopsy. In further studies, we are supposed to focus on molecular mechanisms and signaling pathways in the development of disease so as to promote the biological treatment of cystic angiomatosis.

## Data Availability

All data generated or analyzed during this study are included in this published article.

## Abbreviations

CT: Computed tomography
ALP: Alkaline phosphatase
VEGF: Vascular endothelial growth factor
PET: Positron emission computed tomography
CHEK2: Checkpoint kinase 2
BRIP1: BRCA1 interacting helicase 1
GRM4: Glutamate metabotropic receptor 4
MUC16: Mucin 16
CASC15: Cancer susceptibility 15
CENPF: Centromere protein F
ABCA13: ATP-binding cassette subfamily A member 13
ALK: ALK receptor tyrosine kinase
BLM: BLM RecQ-like helicase
FGFR3: Fibroblast growth factor receptor 3
GO: Gene Ontology
BP: Biological process
CC: Cellular component
MF: Molecular function
GSD: Gorham-Stout disease
